# Nomogram for predicting the biochemical recurrence of prostate cancer after neoadjuvant androgen deprivation therapy

**DOI:** 10.1007/s11255-023-03658-2

**Published:** 2023-06-12

**Authors:** Qi Sun, Yuan-Zhong Yang, Ping Yang, Yong-Hong Li, Yun Cao, Dong Chen, Yijun Zhang

**Affiliations:** 1 State Key Laboratory of Oncology in South China, Collaborative Innovation Center for Cancer Medicine, No. 651, Dongfeng Road East, Guangzhou, 510060 China; 2 Department of Pathology, Sun Yat-sen University Cancer Center, No. 651, Dongfeng Road East, Guangzhou, 510060 China; 3 Department of Urology, Sun Yat-sen University Cancer Center, No. 651, Dongfeng Road East, Guangzhou, 510060 China

**Keywords:** Prostate cancer, nADT, BCR, Predict, Nomogram

## Abstract

**Background:**

A predictive model for biochemical recurrence (BCR) of prostate cancer (PCa) after neoadjuvant androgen deprivation therapy (nADT) has not been established. This study was aimed at determining multiparameter variables that could be used to construct a nomogram to predict the post-nADT BCR of PCa.

**Methods:**

Overall, 43 radical prostatectomy specimens from PCa patients who had undergone nADT were collected. Multiparameter variables were analyzed by univariate and then multivariate logistic analyses to identify the independent prognostic factors for predicting BCR. The predictive model was established using Lasso regression analysis.

**Results:**

Univariate logistic analysis revealed six variables, pathology stage; margins; categorization as group A, B, or C; nucleolus grading; percentage of tumor involvement (PTI); and PTEN status were significantly associated with the BCR of PCa (all p < 0.05). Multivariate logistic regression analysis suggested that categorization as group C, severe nucleolus grading, PTI less than or equal to 5%, and PTEN loss were positively correlated with BCR (all p < 0.05). A nomogram comprising the four variables predicting BCR was constructed, and it exhibited good discrimination (AUC: 0.985; specificity: 86.2%; sensitivity: 100%). Calibration plots for the probability of freedom from BCR at 1 and 2 years showed a good match between the prediction by the nomogram.

**Conclusions:**

We constructed and validated a nomogram to predict the risk of BCR in PCa patients after nADT. This nomogram is a complement to the existing risk stratification systems for PCa, which could have marked implications for clinical decision-making for PCa patients after nADT.

**Supplementary Information:**

The online version contains supplementary material available at 10.1007/s11255-023-03658-2.

## Introduction

Prostate cancer (PCa) is the second most common cancer and the fifth leading cause of cancer-related death in men overall [1]. The use of radical curative treatment for high-risk localized or locally advanced PCa has increased over the past two decades, although previous trials of neoadjuvant androgen-deprivation therapy (nADT) before radical prostatectomy (RP) did not demonstrate an oncological benefit. Interestingly, subset analyses in high-risk PCa patients suggested a trend toward a survival benefit, which aroused controversy among urologists [2]. Tosco et al. showed that the rate of cancer-related death in PCa patients who underwent nADT before RP significantly decreased compared to that in the patients who underwent RP alone [3]. In a prospective phase II study in clinical stage T3 and T4 N0/M0 PCa patients, Berglund et al. showed that the combination of nADT and RP resulted in long-term progression-free survival (PFS) and overall survival (OS) comparable to those achieved with alternative treatment [4]. Recently, James et al. showed that enhanced nADT plus chemotherapy before RP improved the OS in patients with localized high-risk PCa compared with that afforded by RP alone in a phase 3 clinical trial [5]. In clinical practice, nADT before RP is an option in remote areas where surgeons might be inexperienced, in favor of potential tumor remission and improving perioperative safety.

Multiple studies have described the histopathological changes that occur after nADT in PCa patients, including reduced glandular density, decreased glandular diameter, reduction in cytoplasmic quantity, cytoplasmic vacuolization [6–10]. Bernard Têtu et al. first described the histological changes that occur after nADT in PCa patients [6]. Next, Civantos, F and Bullock, M.J. and their colleges confirmed the morphology changes and summarized as decrease in the size and density of neoplastic glands, tumor cells were either vacuolated or had scanty cytoplasm, and immature squamous-cell metaplasia. These changes render the Gleason score of post-therapy specimens nonrepresentative of the disease and, therefore, no longer accurate in assessing disease severity or prognosis [7, 8]. G.Ahlgren et al. found that neuroendocrine (NE) differentiation to be increased in PCa after 3-month nADT treatment, however, the relationship between NE-differentiation and BCR was not mentioned in the study [9]. Recently, Xueli Wang et al. showed that combined pathological indicators in predicting differences in response to nADT in PCa was better than that of model based on individual factor alone [10].

Some studies have proposed meaningful models to assess the prognosis after nADT for patients with PCa. Efstathiou et al. demonstrated that categorization as group A, B, or C based on the pathological morphology of PCa after treatment correlated strongly with the risk of biochemical failure [11]. Murphy et al. proposed using a set of parameters including maximum tumor size, tumor area/volume, cellularity, volume and group A, B, or C for evaluating RPs after neoadjuvant therapy [12]. However, there is no consensus on the pathological evaluation of PCa after nADT for predicting BCR. In this study, we endeavored to construct a nomogram based on clinicopathologic features, molecular markers, and immune microenvironment factors for predicting the BCR of PCa after nADT.

## Materials and methods

### Ethics statement

The Institutional Review Board of Sun Yat-Sen University Cancer Center approved this study.

### Patients

Overall, 43 PCa specimens exposed to nADT and for which complete clinical, pathological, laboratory, and follow-up information was available were collected. The inclusion and exclusion criteria are as follows: (1) Patients who undergo neoadjuvant endocrine therapy for three months or more before radical prostatectomy for prostate cancer. (2) Complete clinical, pathological, laboratory, and follow-up data. (3) Patients with distant metastasis were excluded. The patients had undergone RP and pelvic lymph node dissection after nADT. Pathologic stage and margin status were assigned using the modified American Joint Committee on Cancer staging system. Clinical follow-up information was obtained from the patients’ medical records. BCR was defined as two successive elevations of > 0.2 ng/ml in prostate-specific antigen (PSA) level at least 2 weeks postoperatively.

### Pathological analysis

All specimens were formalin-fixed and paraffin-embedded (FFPE), processed in a routine manner, and stained with hematoxylin and eosin (H&E). Histological slides were reviewed by two pathologists (Q-S. and Y-J.Z.). The morphological changes observed include both parenchymal and interstitial changes, which were described previously [10]. According to the three morphologically distinct groups suggested by Efstathiou et al., group A is defined as small clusters, cords, and isolated TCs; group B is defined as complete, fused small glands; and group C is defined as cribriform growth mode or intraductal spread [11]. Nucleolus grading was performed according to the previously suggested combination of histoarchitectural and cytological grading by Helpap et al.: minimal = 0 points, nuclei: small, round, solitary, homogeneous chromatin; nucleoli: small, solitary, and centrally located; moderate = 1 point, nuclei: size slightly increased, round, solitary, slightly heterogeneous chromatin; nucleoli: slightly enlarged, still solitary, mostly centrally located; and severe = 2 points, nuclei: large, polymorph, heterogeneous chromatin; nucleoli: enlarged, mostly multiple, eccentrically located) [13]. The specimens from RPs were completely submitted and sectioned at 3- to 4-mm intervals with the apical and bladder neck portions sectioned radially to allow for the evaluation of the margin status parallel to the urethra. For each pathological slide, the percentage of the slide with tumor involvement was estimated, and percentage of tumor involvement (PTI) was determined by averaging the estimates from all slides as previously reported [14, 15].

### Immunohistochemistry and scoring

Assays were performed as described previously [16]. The details of the different IHC staining processes are summarized in Supplementary Table 1. The immunohistochemical scores were evaluated by two pathologists (Q-S. and Y-J.Z.). Proliferation (Ki67 index) was estimated semiquantitatively and scored from 0 to 100%. Nuclear staining of any intensity in TCs was considered positive. Androgen receptor (AR) and PTEN expression was observed in the nuclear, and cytoplasmic/nuclear membranes, respectively. According to the staining intensity, the TCs were divided into two categories: positive, showing staining intensity; and negative, showing a complete absence of staining. Neuroendocrine marker expression (NME) was defined as either CD56- or Syn-positive expression. CD56- or Syn-positive expression was defined as staining intensity in the cytomembrane or cytoplasm, respectively.

IHC was also applied to evaluate the immunophenotype of TCs and tumor-infiltrating lymphocytes. PD-L1 expression in TCs and infiltrating immune cells (ICs) and PD1 expression in ICs were evaluated for every case. Positive expression was defined as tumor or lymphoid cells that demonstrated at least partially weak to strong expression in IHC. The average density of TCs (cells/high-power field [HPF]) was determined based on CD8, FoxP3, CD163, and CD68 cells, and three fields of view (magnification × 400) in tumor tissue areas were randomly selected and counted to determine the absolute number of cells with positive staining; subsequently, the average number of cells was determined. To determine the percentage, a representative section for the entire tumor area was evaluated.

### Statistical analysis

Statistical analyses were performed using IBM SPSS Statistical software version 26.0 (IBM Corp., Chicago, IL, USA) and R version 3.6.0 (http://www.R-project.org). The optimal cut-off values of related parameters were all transformed into categorical variables based on the cut-off values determined using the R package “pROC” [17]. Differences in distribution between patients in the non-BCR and BCR cohorts were analyzed using the Chi-square test. Wilcoxon rank-sum or Fisher’s exact test was used for categorical variables, and the t-test was used for discrete variables. Lasso regression analysis was used to select the most useful prognostic variables in the cohort. According to the regulation weight λ, LASSO shrinks all regression coefficients toward zero and sets the coefficients of many irrelevant features to zero. The optimal values of the penalty parameter λ were determined by tenfold cross-validation with one standard error of the minimum criteria (1-SE criteria), where the final value of λ yielded a minimum cross-validation error. Retained features with nonzero coefficients were used for regression model fitting [18, 19]. Next, a prognostic computing-based model was established for each patient through a linear combination of selected variables weighted by their respective coefficients. The R package “glmnet” was used for Lasso regression analysis. The area under the curve (AUC) was calculated using the “pROC” package. Model performance was assessed by plotting a calibration curve in internal validation with bootstrapping (1000 bootstrap resamples) [20].

## Results

### Descriptive clinical characteristics of the cohort

In all, 43 cases of PCa after nADT were included, of which 14 (32.6%) cases showed evidence of postoperative BCR. The median follow-up period, BCR time, and patient age were 23.6 months (range 1.5–67.7 months), 24.9 months (range 1.5–50.7 months), and 68 years (range 50–82 years), respectively. The median pretreatment PSA level, preoperative serum PSA level, and preoperative fPSA/tPSA were 49.89 (range 1.63–400 ng/mL), 0.226 (range 0.003–24.51 ng/mL), and 0.14 (range 0.01–1.43), respectively. No significant association was identified between these clinical characteristics and BCR (Supplementary Table 2).

### Pathological characteristics of the cohort

**Fig. 1:**
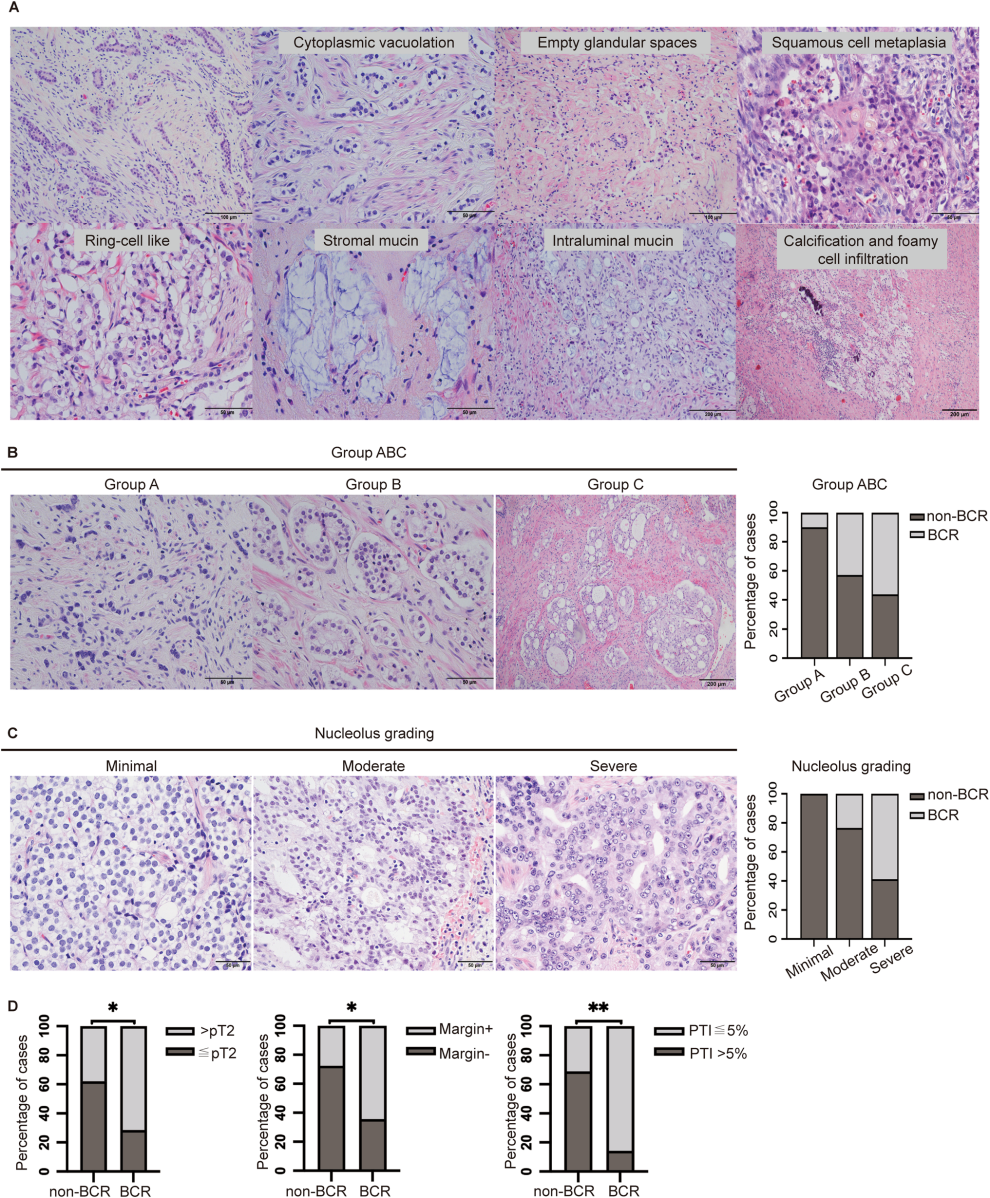
The representative morphology of preoperatively treated prostate cancer and the correlation of pathological parameters with biochemical recurrence. **A** The representative morphology of prostate cancer after neoadjuvant androgen deprivation therapy (nADT). **B** The representative morphology of group A, B, and C, and the association with biochemical recurrence (BCR). **C** The representative morphology of the nucleolus and the association with BCR. **D** Pathology stage, margin, and percentage of tumor involvement (PTI) were associated with BCR (*p < 0.05; **p < 0.01)

The histologic evaluation of the RP specimens showed that the morphological changes after nADT described before also occurred in our cases, including reduced glandular density, decreased glandular diameter, reduced cytoplasmic quantity, cytoplasmic vacuolation, nuclear pyknosis, apoptosis, squamous cell metaplasia, interstitial changes in stromal mucin, calcification, foamy cell infiltration, and stromal increase (Fig. 1A). Regarding the three morphologically distinct groups, BCR was more frequently associated with group C than with groups A (2/14) and B (3/14) (groups A, B, and C = 14.3%, 21.4%, and 64.3%, respectively; p = 0.003) (Fig. 1B). Regarding nucleolus grading, the severe group (10/14) was more commonly associated with BCR than the minimal (0/14) and moderate groups (4/14) (minimal, moderate, and severe = 0%, 28.6%, and 71.4%,respectively; p = 0.002) (Fig. 1C). Additionally, greater than pT2 was more frequently observed in the BCR group (10/14) than non BCR group (11/29) (71.4% vs 37.9%, p = 0.039). 64.3% (9/14) BCR patients showed margin positive, but only 27.6% (8/29) cases had margin negative (p = 0.021). We identified 85.7% (12/14) BCR cases with PTI greater than 5%, whereas 31.1% (9/29) non-BCR case with PTI less than or equal to 5% (p = 0.001) (Fig. 1D). However, there is no significant association between seminal invasion, lymph node invasion, vascular invasion, perineural invasion, tumor diameter, and BCR (Supplementary Table 2).

### Molecular marker parameters of the cohort

**Fig. 2:**
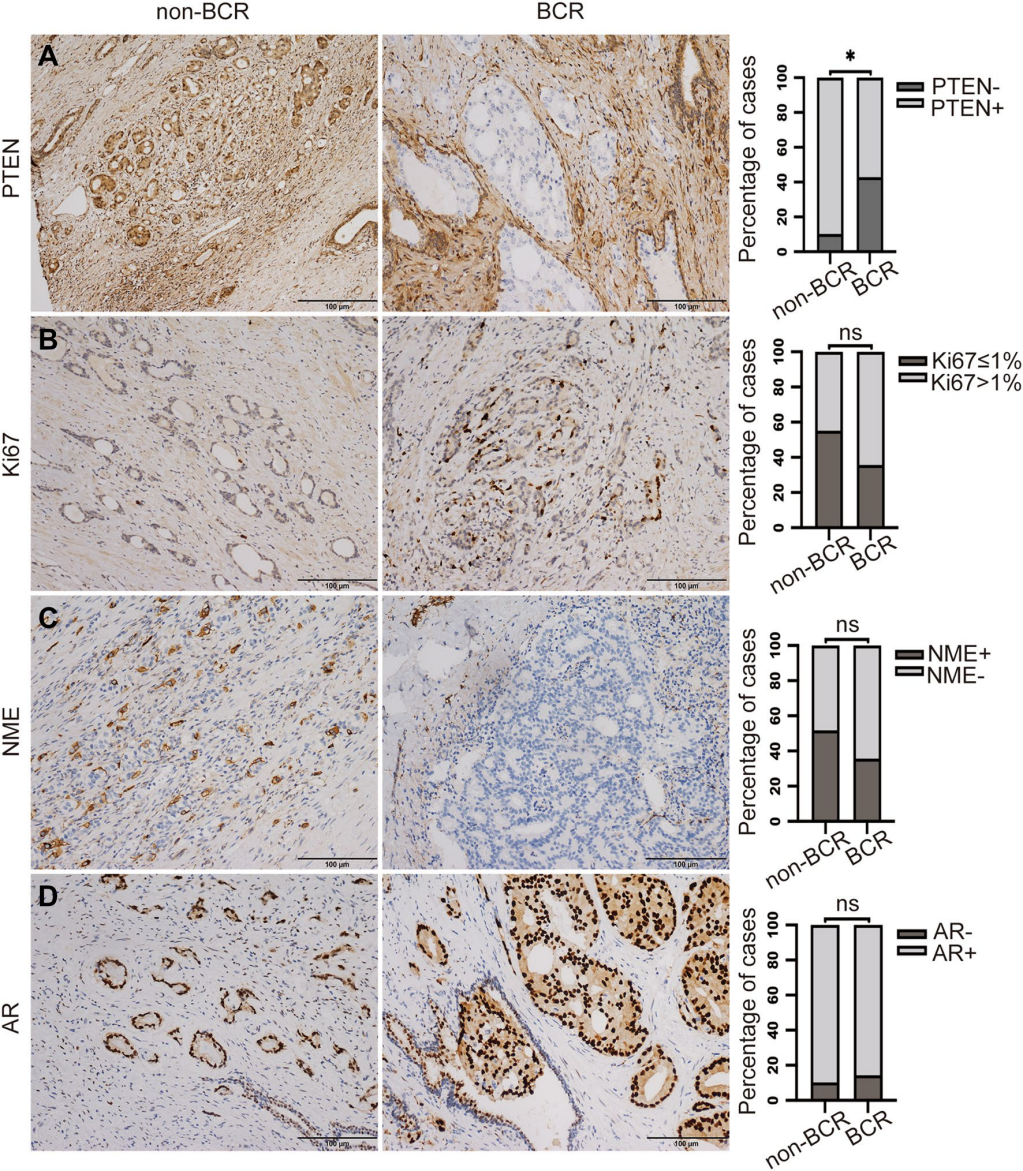
The correlation of molecular marker parameters with biochemical recurrence. **A** PTEN loss was significantly correlated with biochemical recurrence (BCR) (p = 0.014). (B/C/D) Ki67 index, neuroendocrine marker expression (NME), and androgen receptor (AR) expression were not correlated with BCR (*p < 0.05; ns = no significance)

Immunophenotypically, 88.4% (38/43) cases showed AR positive expression and 20.9% (9/43) cases showed PTEN loss. 51.2% (22/43) cases showed Ki67 index greater than 1% and 46.5% (20/43) cases showed NME positive (Supplementary Table 3). The results demonstrated PTEN loss was significantly correlated with BCR (p = 0.014, odds ratio [OR] = 0.154, 95% confidence interval [CI] 0.031–0.759), while AR, Ki67 index and NME were all not correlated with BCR (Fig. 2).

### Immune microenvironment parameters of the cohort

**Fig. 3:**
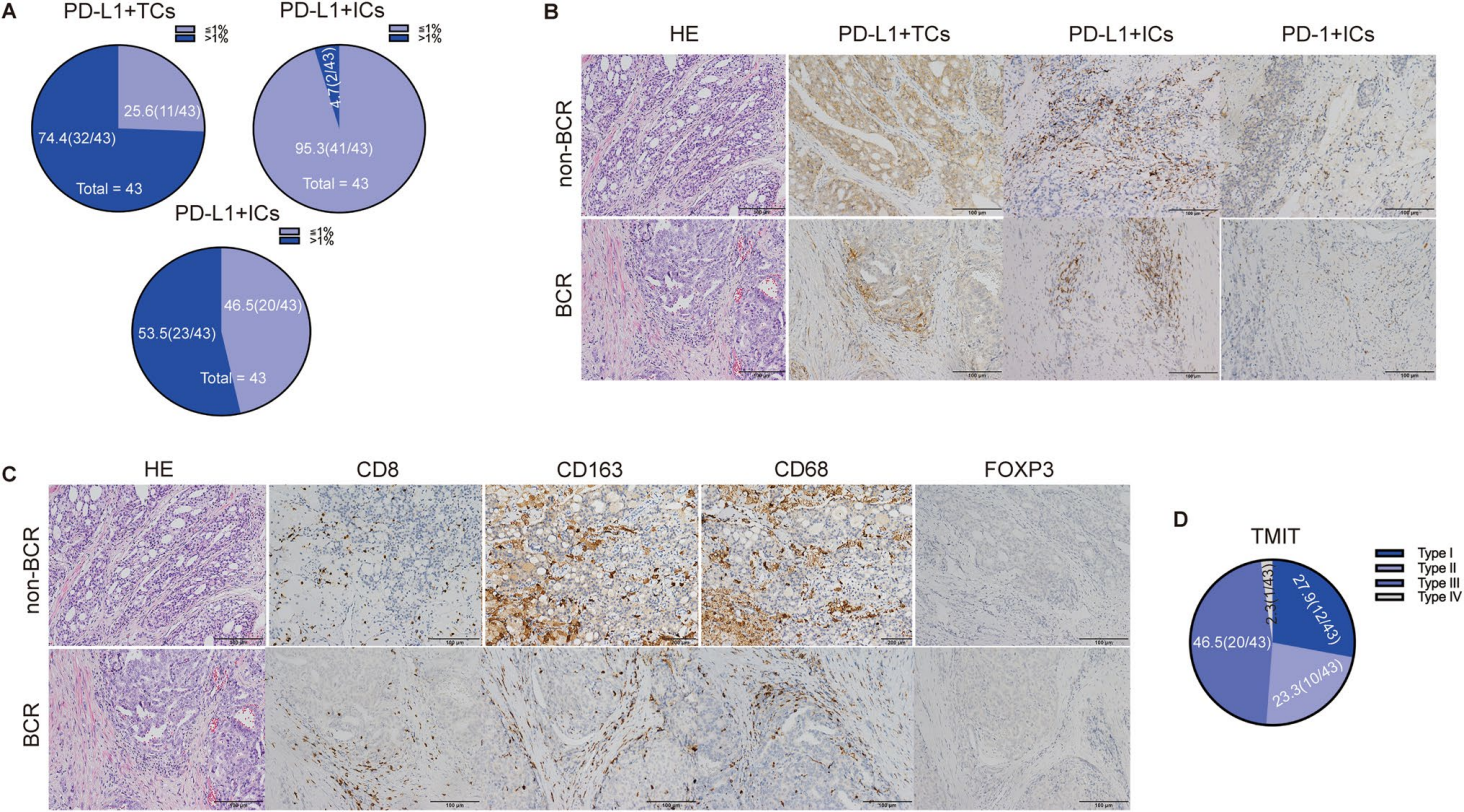
The correlation of immune microenvironment parameters with biochemical recurrence. **A** PD-L1 + TCs, PD-L1 + ICs, and PD1 + ICs in 43 prostate cancer patients based on IHC staining. **B** Representative morphology of the PD-L1 and PD1 expression in non-biochemical recurrence (BCR) and BCR, respectively. **C** Representative morphology of CD8, FOXP3, CD68, CD163 in 43 prostate cancer patients based on IHC staining. **D** Tumor microenvironment immune types (TMIT) in 43 prostate cancer patients based on IHC staining

PD-L1 was expressed in > 1% of TCs in 74.4% (32/43) of the patients and in > 1% ICs in 4.7% (2/43) of the patients. PD-1 was expressed in > 1% of ICs in 53.5% (23/43) of the patients (Fig. 3A). No significant differences were identified between PD-L1, PD-1 and BCR (Fig. 3B and Supplementary Table 5). Other immunoenvironment parameters such as FOXP3, CD8, CD68, and CD163 has no correlate with BCR after nADT in PCa patients (Fig. 3C and Supplementary Table 4). According to the previous definition [21], tumor microenvironment immune types (TMIT) were as follows: TMIT I (PD-L1 + /CD8^High^) = 12 samples (27.9%); TMIT II (PD-L1 − /CD8^Low^) = 10 samples (23.3%); TMIT III (PD-L1 + /CD8^Low^) = 20 samples (46.5%), and TMIT IV (PD-L1 − /CD8^High^) = 1 sample (2.3%) (Fig. 3D and Suplementary Table 5).

### Construction of the multiparametric model

**Fig. 4:**
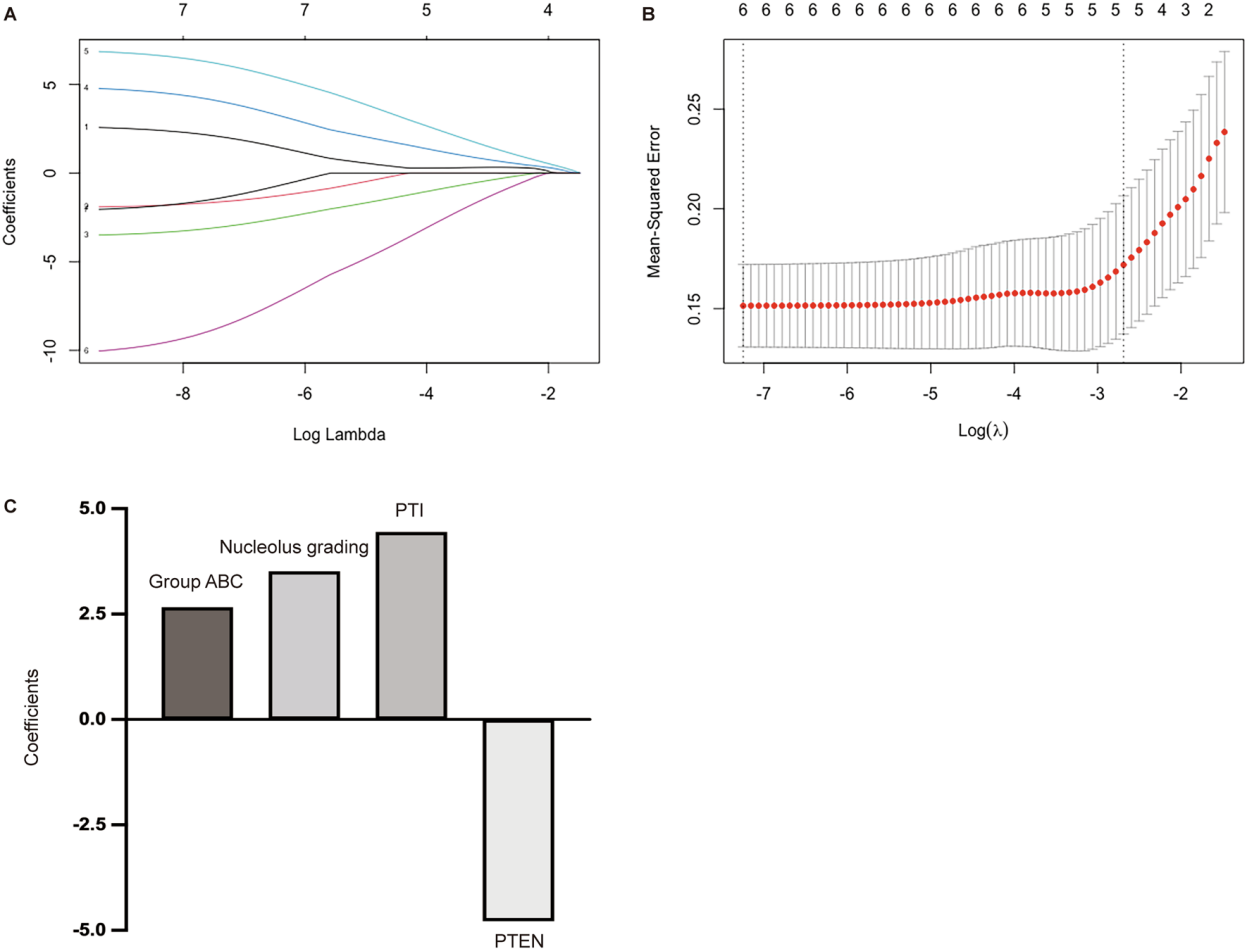
Potential predictor selection using Lasso regression analysis. **A** The changing trajectory of each predictor. The horizontal axis represents the log value of each predictor λ, and the vertical axis represents the coefficient of the independent predictor. **B** Tenfold cross-validation was used for model establishment, and the confidence interval under each λ value. **C** Histogram shows the role of each predictor that contribute to the developed prognostic model. The predictors that contribute to the prognostic model are plotted on the x-axis, with their coefficients in the Lasso regression analysis plotted on the y-axis

To select the parameters predicting BCR after nADT in PCa patients, the parameters that were associated with BCR in the univariate analysis, such as categorization as group A, B, or C; nucleolus grading; PTI; PTEN; margin; and pathologic stage, were subjected to Lasso regression analysis. Figure 4A shows the change in trajectory for each factor analyzed. The optimal value of λ was 0.05949808 in the Lasso regression analysis (Fig. 4B). Thus, this value was selected for the final model, including four predictors from the six parameters that were significantly weighted prognostic factors: categorization as group A, B, or C; nucleolus grading; PTI; and PTEN. The coefficients of the 4 predictors are presented in Fig. 4C. Next, a predicted model risk score was calculated based on the personalized levels of the four predictors, by using the following formula: prediction of BCR risk score =  − 10.096 + (2.662 × group A, B, or C) + (3.514 × nucleolus grading) + (4.447 × PTI) − (4.785 × PTEN). In this formula, each variable level was valued as 0 or 1. A value of 0 was assigned when the marker was less than or equal to the corresponding cut-off value; otherwise, a value of 1 was assigned [22].

### Construction and verification of the nomogram model

**Fig. 5:**
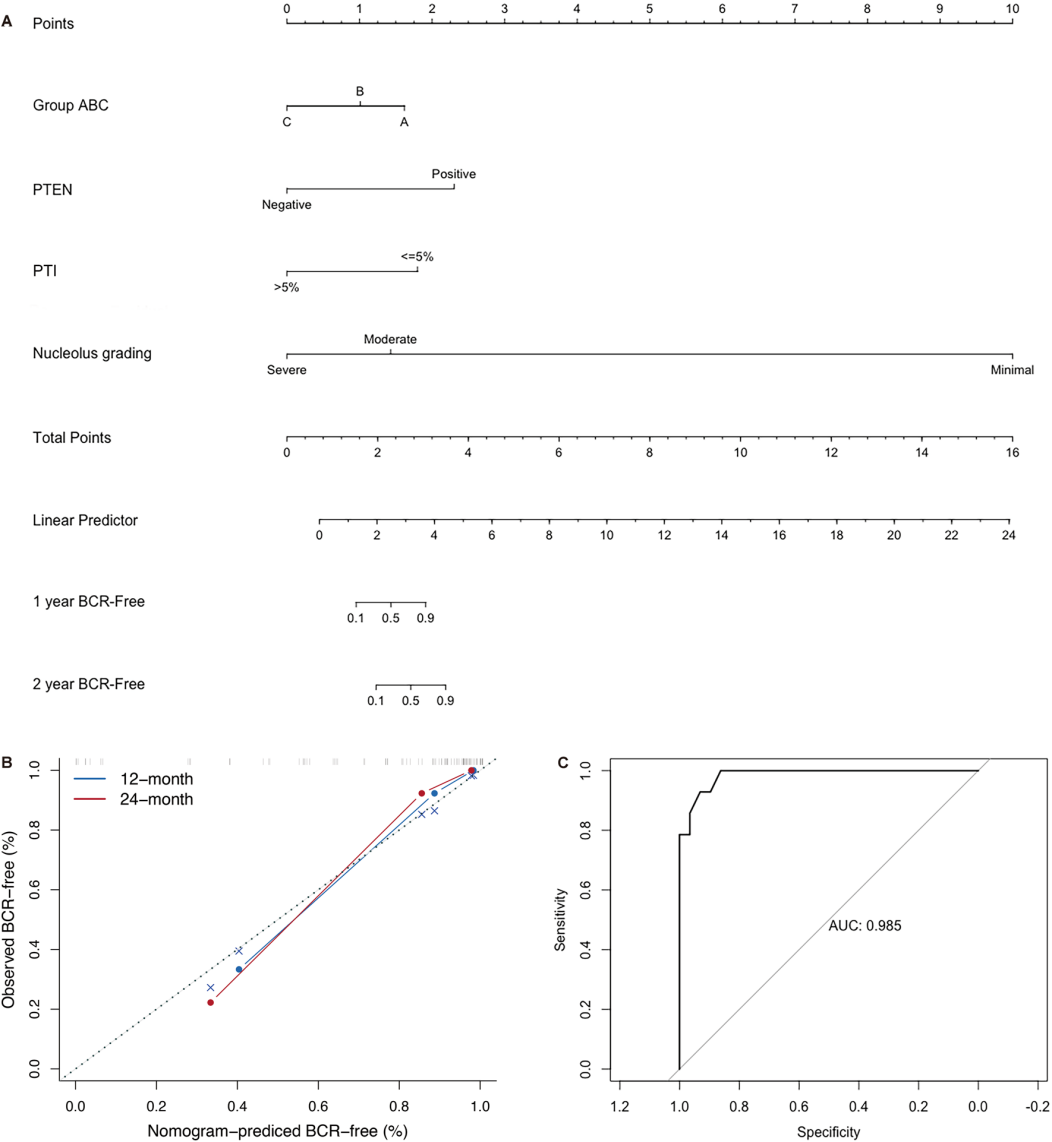
The nomogram and evaluation of the model’s performance. **A** The nomogram: A nomogram comprised group A, B, or C; PTEN status, percentage of tumor involvement (PTI), and nucleolus grading to predict the 12- and 24-month BCR-free probability. **B** The calibration: Calibration of the nomogram at the 12- and 24-month endpoints. **C** The ROC curve of the nomogram: Receiver operating characteristic (ROC) curve and area under the curve (AUC) for evaluating the predictive accuracy of the nomogram

A multivariable analysis based on logistic regression was performed to construct a prediction model to estimate the probability of BCR. The significant parameters associated with BCR in this model included categorization as group A, B, or C (p = 0.044, OR = 14.331, 95% CI 1.918–59.881); nucleolus grading (p = 0.009, OR = 33.595, 95% CI 4.138–118.945); PTI (p = 0.049, OR = 85.408, 95% CI 2.795–545.443); and PTEN (p = 0.048, OR = 0.008, 95% CI 0.006–0.292). The nomogram was graphically depicted based on these results (Fig. 5A). Calibration plots for the probability of freedom from BCR at 1 and 2 years showed a good match between the prediction by the nomogram (Fig. 5B). The area under the ROC curves (AUCs) of the predicted model was 0.985 (95% CI: 0.9478–1), indicating a good ability to predict BCR (Fig. 5C).

## Discussion

In this study, we constructed a nomogram based on clinicopathologic features, molecular markers, and immune microenvironment parameters to provide a theoretical basis for clinical decisions. The key parameters included categorization as group A, B, or C; nucleolus grading; PTI; and PTEN status.

The Gleason grading system remains one of the most useful prognostic predictors in PCa [23]. However, most pathologists now recommend not using the Gleason score following nADT because of the lack of adapted criteria to grade these tumors, poor reproducibility, and the lack of biological and clinical relevance of the grading after hormone manipulation. Neil et al. categorized pretreated PCa into three morphologically distinct groups based on hierarchical clustering analysis [11]. We found that categorization as group A, B, or C was an independent predictor of BCR and was therefore considered in the construction of the nomogram. Additionally, we found that group C was more commonly associated with BCR compared with groups A and B. This result is consistent with those of previous studies [11, 24, 25]. Thus, categorization as group A, B, or C is a potential prognostic risk stratification criterion.

Interestingly, nucleolus grading had a better predictive effect than did other prognostic factors. After neoadjuvant therapy, TCs may exhibit nuclear pyknosis; however, some TCs would still show enlargement, multiple nucleoli, and heterogeneity of chromatin, which are associated with poor prognoses. We flexibly applied a nucleolus grading system, which was confirmed to correlate with the prognosis of PCa patients by Helpap et al. [13], to be used in the assessment of PCa cells after nADT. Our results showed that BCR was more common in the severe group compared with that in the minimal and moderate groups. The result highlighted the post-treatment tumor growth state in terms of cell morphology.

The identification of surrogates for survival end points in high-risk PCa is of utmost importance [26]. There is still considerable controversy regarding the evaluation of minimum residual disease (MRD) after nADT because it is difficult to measure the greatest dimension of a tumor, owing to it being distributed unevenly within the residual tumor bed as scattered islands of residual disease. Some studies defined MRD as a tumor of size < 0.5 cm in the RP specimen; others defined MRD as a residual cancer burden of < 0.25 cm^3^ [27–30]. The PTI, which was determined by averaging the percentage tumor involvement in the whole prostate, solved the problem of the controversy of MRD and is an independent prognostic factor of BCR.

PTEN is a key tumor suppressor gene in PCa [31]. Deletion of PTEN occurs in 20–70% of PCa patients and has been linked to rapid tumor progression and early recurrence. PTEN loss after nADT was observed in 20.9% of PCa cases; moreover, it is associated with a worse prognosis [32–35]. PTEN status could potentially improve current risk stratification protocols when the Gleason score is inaccurate. In this study, PTEN status had a high positive predictive value (67%) and negative predictive value (77%) in predicting BCR.

We constructed a multiparametric nomogram to predict the risk of BCR after nADT in PCa patients. This nomogram is a complement to the existing risk stratification systems for PCa, which could have significant implications for clinical decision-making after nADT in PCa patients.

## Supplementary Information

Below is the link to the electronic supplementary material.

Supplementary file1 (DOCX 31 KB)

## Data Availability

The datasets analyzed in the current study are not publicly available due to patient privacy concerns but are available from the corresponding author on reasonable request.
