# Supplementary material for: Nomogram for predicting the biochemical recurrence of prostate cancer after neoadjuvant androgen deprivation therapy
Source: Int Urol Nephrol. 2023 Jun 12;55(9):2215–24. doi: 10.1007/s11255-023-03658-2 (PMC10406657; doi:10.1007/s11255-023-03658-2)
Supplement: Supplementary file 1 — Supplementary file1 (DOCX 31 KB) [file 11255_2023_3658_MOESM1_ESM.docx]

**Supplementary Tables**

**Table 1. Antibody characteristics and protocol for whole-tissue immunohistochemistry.**

| Marker | Principal Role | Catalog Number | Company | Dilution | Positive Control | Cellular Localization |
| --- | --- | --- | --- | --- | --- | --- |
| CD8 | Cytotoxic T cell | ZA-0508 | ZSJQ-BIO | 1：200 | Tonsil | Membrane/cytoplasm |
| FOXP3 | Regulatory T cell | AB215206 | ABCAM | 1：100 | Tonsil | Nucleus |
| CD68 | Macrophage | ZM-0060 | ZSJQ-BIO | 1：200 | Tonsil | Cytoplasm |
| CD163 | M2 macrophage | ZM-0428 | ZSJQ-BIO | 1：200 | Tonsil | Cytoplasm/membrane |
| PD1 | Immune checkpoint | ZM-0381 | ZSJQ-BIO | 1：100 | Tonsil | Cytoplasm/membrane |
| PD-L1 | Immune checkpoint | 13684(E1L3N) | Cell Signaling | 1：100 | Tonsil | Membrane/cytoplasm |
| Syn | Neuroendocrine differetiation | ZA-0506 | ZSJQ-BIO | 1：150 | Tonsil | Cytoplasm |
| CD56 | Neuroendocrine differetiation | ZM-0057 | ZSJQ-BIO | 1：150 | Tonsil | Membrane |
| AR | Androgen expression | ZA-0554 | ZSJQ-BIO | 1：100 | Tonsil | Nucleus |
| PTEN | Prognosis related | 13866 | Cell Signaling | 1：100 | Tonsil | Nucleus/cytoplasm |
| Ki67 | Prognosis related | ZM-0166 | ZSJQ-BIO | 1：100 | Tonsil | Nucleus |

**Table 2. The clinico-pathological characteristics in the patients**

| Characteristics | Overall (n=43) | non-BCR (n=29) | BCR (n=14) | P Value |
| --- | --- | --- | --- | --- |
| Age | 68 (50-82) | 68 (50-79) | 66 (53-82) | 0.343 |
| Serum PSA (ng/mL) at diagnosis |  | | | 0.493 |
| 0-4 | 2 (4.7) | 1 (3.4) | 1 (7.1) |  |
| 4.1-10 | 1 (2.3) | 0 (0) | 1 (7.1) |  |
| 10.1-20 | 4 (9.3) | 3 (10.3) | 1 (7.1) |  |
| ＞20 | 36 (83.7) | 25 (86.3) | 11 (78.7) |  |
| Preoperative PSA (ng/mL) |  | | | 0.150 |
| Median (range) | 0.226 | 0.198 (0.003-10.45) | 0.291 (0.021-24.51) |  |
| Preoperative fPSA/tPSA |  | | | 0.492 |
| Median (range) | 0.14 | 0.16 (0.01-0.91) | 0.11 (0.05-1.43) |  |
| Clinical T stage |  | | | 0.287 |
| T2 | 20 (46.5) | 15 (51.7) | 5 (35.7) |  |
| T3 | 14 (32.6) | 9 (31.1) | 5 (35.7) |  |
| T4 | 9 (20.9) | 5 (17.2) | 4 (28.6) |  |
| Gleason Score |  |  |  | 0.813 |
| 3+4 | 9 (20.9) | 6 (20.7) | 3 (21.4) |  |
| 4+3 | 8 (18.6) | 5 (17.2) | 3 (21.4) |  |
| >7 | 26 (60.5) | 18 (62.1) | 8 (57.2) |  |
| Pathology stage |  |  |  | 0.039 |
| ≦pT2 | 22 (51.2) | 18 (62.1) | 4 (28.6) |  |
| >pT2 | 21 (48.8) | 11 (37.9) | 10 (71.4) |  |
| Margin |  |  |  | 0.021 |
| Negative | 26 (60.5) | 21 (72.4) | 5 (35.7) |  |
| Positive | 17 (39.5) | 8 (27.6) | 9 (64.3) |  |
| Seminal invasion |  |  |  | 0.757 |
| Negative | 26 (60.5) | 18 (62.1) | 8 (57.1) |  |
| Positive | 17 (39.5) | 11 (37.9) | 6 (42.9) |  |
| Lymph node invasion |  |  |  | 0.665 |
| Negative | 32 (74.4) | 21 (72.4) | 11 (78.8) |  |
| Positive | 11 (25.6) | 8 (27.6) | 3 (21.2) |  |
| Vascular invasion |  |  |  | 0.937 |
| Negative | 28 (65.1) | 19 (65.5) | 9 (64.3) |  |
| Positive | 15 (34.9) | 10 (34.5) | 5 (35.7) |  |
| Perineural invasion |  |  |  | 0.946 |
| Negative | 12 (27.9) | 8 (27.6) | 4 (28.6) |  |
| Positive | 31 (72.1) | 21 (72.4) | 10 (71.4) |  |
| ABC Group |  |  |  | 0.003 |
| A | 20 (46.5) | 18 (62.1) | 2 (14.3) |  |
| B | 7 (16.3) | 4 (13.8) | 3 (21.4) |  |
| C | 16 (37.2) | 7 (24.1) | 9 (64.3) |  |
| Nucleolus grading |  |  |  | 0.007 |
| Minimal | 9 (21.0) | 9 (31.1) | 0 (0) |  |
| Moderate | 17 (39.5) | 13 (44.8) | 4 (28.6) |  |
| Severe | 17 (39.5) | 7 (24.1) | 10 (71.4) |  |
| PTI |  |  |  | 0.001 |
| ≦5% | 22 (51.2) | 20 (68.9) | 2 (14.3) |  |
| >5% | 21 (48.8) | 9 (31.1) | 12 (85.7) |  |

PTI = percentage of tumor involvement

**Table 3.** **Prognostic parameters in the patients**

| Immunological Markers | Overall (n=43) | non-BCR (n=29) | BCR (n=14) | p-Value |
| --- | --- | --- | --- | --- |
| AR |  |  |  | 0.706 |
| Negative | 5 (11.6) | 3 (10.3) | 2 (14.3) |  |
| Positive | 38 (88.4) | 26 (89.7) | 12 (85.7) |  |
| PTEN |  |  |  | 0.014 |
| Negative | 9 (20.9) | 3 (10.3) | 6 (42.8) |  |
| Positive | 34 (79.1) | 26 (89.7) | 8 (57.2) |  |
| Ki67 index |  |  |  | 0.322 |
| ≦1% | 21 (48.8) | 16 (55.2) | 5 (35.7) |  |
| > 1% | 22 (51.2) | 13 (44.8) | 9 (64.3) |  |
| NME |  |  |  | 0.324 |
| Negative | 23 (53.5) | 14 (48.3) | 9 (64.3) |  |
| Positive | 20 (46.5) | 15 (51.7) | 5 (35.7) |  |

NME = neuroendocrine marker expression

**Table 4. Immune microenvironment parameters in the patients**

| Immunological Markers | Overall (n=43) | non-BCR (n=29) | | BCR (n=14) | | p-Value |
| --- | --- | --- | --- | --- | --- | --- |
| PD-L1(TPS) |  |  | | |  | 0.665 |
| ≦1% | 11 (25.6) | 8 (27.6) | | | 3 (21.4) |  |
| > 1% | 32 (74.4) | 21 (72.4) | | | 11 (78.6) |  |
| PD-L1(IPS) |  | |  | |  | 0.474 |
| ≦1% | 41 (95.3) | 27 (93.1) | | | 14 (100.0) |  |
| > 1% | 2 (4.7) | 2 (6.9) | | | 0 (0) |  |
| PD1(/HPF) |  |  | | |  | 0.446 |
| ≦2 | 15 (34.9) | 9 (31.1) | | | 6 (42.9) |  |
| > 2 | 28 (65.1) | 20 (68.9) | | | 8 (57.1) |  |
| PD1 proportion |  |  | | |  | 0.331 |
| ≦1% | 20 (46.5) | 12 (41.4) | | | 8 (57.1) |  |
| > 1% | 23 (53.5) | 17 (58.6) | | | 6 (42.9) |  |
| FOXP3(/HPF) |  |  | | |  | 0.054 |
| ≦1 | 32 (74.4) | 19 (65.5) | | | 13 (92.9) |  |
| > 1 | 11 (25.6) | 10 (34.5) | | | 1 (7.1) |  |
| FOXP3 proportion |  |  | | |  | 0.054 |
| ≦1% | 32 (74.4) | 19 (65.5) | | | 13 (92.9) |  |
| > 1% | 11 (25.6) | 10 (34.5) | | | 1 (7.1) |  |
| CD8(/HPF) |  |  | | |  | 0.232 |
| ≦45 | 22 (51.2) | 13 (44.8) | | | 9 (64.3) |  |
| > 45 | 21 (48.8) | 16 (55.2) | | | 5 (35.7) |  |
| CD8 proportion |  |  | | |  | 0.114 |
| ≦45% | 30 (69.8) | 18 (62.1) | | | 12 (85.7) |  |
| > 45% | 13 (30.2) | 11 (37.9) | | | 2 (14.3) |  |
| CD68(/HPF) |  |  | | |  | 0.212 |
| ≦17 | 3 (6.9) | 3 (10.3) | | | 0 (0) |  |
| > 17 | 40 (93.1) | 26 (89.7) | | | 14 (100.0) |  |
| CD68 proportion |  |  | | |  | 0.059 |
| ≦25% | 18 (41.9) | 15 (51.7) | | | 3 (21.4) |  |
| > 25% | 25 (58.1) | 14 (48.3) | | | 11 (78.6) |  |
| CD163(/HPF) |  |  | | |  | 0.129 |
| ≦95 | 31 (72.1) | 23 (79.3) | | | 8 (57.1) |  |
| > 95 | 12 (27.9) | 6 (20.7) | | | 6 (42.9) |  |
| CD163 proportion |  |  | | |  | 0.098 |
| ≦35% | 5 (11.6) | 5 (17.2) | | | 0 (0) |  |
| > 35% | 38 (88.4) | 24 (82.8) | | | 14 (100.0) |  |

**Table 5. Tumor microenvironment immune types (TMIT) of the patients**

| TMIT | Overall (n=43) | non-BCR (n=29) | BCR (n=14) | p-Value |
| --- | --- | --- | --- | --- |
|  |  |  |  | 0.168 |
| Type I | 12 (27.9) | 10 (34.5) | 2 (14.3) |  |
| Type II | 10 (23.3) | 7 (24.1) | 3 (21.4) |  |
| Type III | 20 (46.5) | 11 (37.9) | 9 (64.3) |  |
| Type IV | 1 (2.3) | 1 (3.5) | 0 (0) |  |
